# General circulation and global heat transport in a quadrupling CO_2_ pulse experiment

**DOI:** 10.1038/s41598-022-15905-0

**Published:** 2022-07-07

**Authors:** Soon-Il An, So-Eun Park, Jongsoo Shin, Young-Min Yang, Sang-Wook Yeh, Seok-Woo Son, Jong-Seong Kug

**Affiliations:** 1 Department of Atmospheric Sciences and Irreversible Climate Change Research Center, Yonsei University, Seodaemun-gu, Seoul, 03722 Republic of Korea; 2 Division of Environmental Science and Engineering, Pohang University of Science and Technology (POSTECH), Pohang, Republic of Korea; 3 Department of Atmospheric Science, Key Laboratory of Meteorological Disaster of Ministry of Education, Joint International Research Laboratory of Climate and Environment Change, Collaborative Innovation Center on Forecast and Evaluation of Meteorological Disasters and Earth System Modeling Center, Nanjing University of Information Science and Technology, Nanjing, 210044 China; 4 Department of Atmospheric Sciences and International Pacific Research Center, University of Hawaii, Honolulu, HI 96822 USA; 5 Marine Science and Convergence Engineering, Hanyang University, ERICA, Ansan, Republic of Korea; 6 School of Earth and Environmental Sciences, Seoul National University, Seoul, Republic of Korea; 7 Institute for Convergence Research and Education in Advanced Technology, Yonsei University, Seoul, 03722 Republic of Korea

**Keywords:** Atmospheric dynamics, Physical oceanography

## Abstract

To investigate the response of the general circulation and global transport of heat through both atmosphere and ocean to two-types of carbon dioxide removal scenario, we performed an earth system model experiment in which we imposed a pulse-type quadrupling of CO_2_ forcing for 50 years and a gradual peak-and-decline of four-time CO_2_ forcing. We found that the results from two experiments are qualitatively similar to each other. During the forcing-on period, a dominant warming in the upper troposphere over the tropics and on the surface at high latitudes led to a slowdown in the Hadley circulation, but the poleward atmospheric energy transport was enhanced due to an increase in specific humidity. This counteracted the reduction in poleward oceanic energy transport owing to the suppression of the meridional overturning circulation in both Hemispheres. After returning the original CO_2_ level, the hemispheric thermal contrast was reversed, causing a southward shift of the intertropical convergence zone. To reduce the hemispheric thermal contrast, the northward energy transports in the atmosphere and ocean surface were enhanced while further weakening of the global-scale Atlantic meridional overturning circulation led to southward energy transport in the deep ocean.

## Introduction

Since the Industrial Revolution, the climate of the Earth has undergone unprecedented changes. Such climate change is caused by high and abrupt emissions of greenhouse gases^1^. Fortunately, the Paris Agreement of December 2015, i.e., the United Nations agreement on maintaining the increase in global average temperature at well below 2 °C from pre-industrial levels and that efforts to limit the temperature increase to 1.5 °C should be pursued, provides hope that the worst scenario for future climate change can be avoided. However, scientific and more quantitatively accurate projections of future climate change, in terms of spatiotemporal aspects, are required to mitigate the climate crisis in the most efficient manner.

Future projections can be obtained from the state-of-the-art general circulation model simulations under various scenarios, particularly the Coupled Model Intercomparison Project phase 5 and 6 (CMIP5, 6)^2–4^. One such projection is the so-called “abrupt4 × CO_2_” experiment endorsed in CMIP5, in which atmospheric carbon dioxide (CO_2_) is instantaneously quadrupled and then kept constant for 150 years. Such a step-function type of changes in CO_2_ levels may not be realistic, but does have an advantage in understanding the fundamental behavior of climate system than more complicated scenarios could do. Good et al.^5^ revealed that the climate behavior illustrated by the “abrupt4 × CO_2_” simulations is also applicable to more realistic projections such as the representative concentration pathway scenarios, indicating that the “abrupt4 × CO_2_” simulations can be used to help make future projections computationally less expensive^6,7^.

As an extension of previous studies, this work examines the changes in atmospheric and oceanic general circulation when CO_2_ levels are abruptly not only increased but also decreased. We used a “4 × CO_2_pulse” experiment, in which the atmospheric CO_2_ concentration was instantaneously quadrupled from a pre-industrial (PI) level and maintained as constant for 50 years. The CO_2_ level was then instantaneously reduced to the PI level. This experiment is similar to the “abrupt4 × CO_2_” experiment in the warming stage but differs in that it also considers an abrupt decrease in CO_2_. Such a change is more analogous to the carbon dioxide removal model intercomparison project (CDRMIP) endorsed in CMIP6^8^. In CDRMIP experiment, atmospheric CO_2_ concentrations are gradually increased by 1% per year until they reach four times the initial level and then decreased toward the initial level in a mirror pathway. In this study, we investigated how closely the global and regional climate adjustments in the 4 × CO_2_pulse experiment—with its abrupt change in CO_2_ levels—resembled those in the CDRMIP experiment with a gradual CO_2_ change.

The aforementioned studies^5–7^ focused mainly on the global mean surface air temperature (GMST) and global mean oceanic heat uptake. These two physical quantities are key metrics of climate change projections, but regional climate change must be substantially more informative to human life. The GMST can be stabilized through a balance between incoming solar energy and outgoing terrestrial radiative energy. However, stabilizing regional climates also requires dynamic energy transport to reduce the heating imbalance between net surplus radiative energy and net loss radiative energy. Therefore, to address regional climate change associated with global warming, both local radiative energy balances and the energy transfer processes should be fully understood.

As representative regional responses to greenhouse gas forcing, polar amplification^9,10^ and the migration of the Intertropical Convergence Zone^11^ (ITCZ) are known to be strongly influenced by the meridional atmospheric heat transport (AHT). Likewise, local sea surface temperature (SST) responses to greenhouse gas forcing is determined by surface energy exchanges with the atmosphere, oceanic heat uptake into the deep ocean, and oceanic heat transport (OHT). Therefore, to understand a process related to regional climate change, it is necessary to investigate how atmospheric and oceanic general circulation and heat transport will respond to changing CO_2_ forcing. Previous studies have shown that when CO_2_ levels quadruple, as modeled in CMIP5, poleward OHT decreases, possibly due to a weakened Atlantic meridional overturning circulation^12^ (AMOC), but AHT increases by an amount that nearly compensates for this^13^. Such compensation could be interpreted as a climate-state invariance of total meridional heat transport (MHT; MHT = AHT + OHT) in a changing climate, i.e., the so-called Bjerknes Compensation^12,14–16^ (BJC). However, other studies argued that the climate-state invariance in MHT could be imperfect due to changes in sea ice cover^15^ or cloud cover^17^. Furthermore, the BJC may be time scale-dependent^18^ such that as the time scale increases, the BJC could reach an equilibrium^16^. This suggests that BJC could also be influenced by the time scale of forcing. So far, AHT and OHT have been documented for a warming trend but are not well documented for a cooling trend. The 4 × CO_2_pulse experiment conducted for this study will provide insights into understanding the changes in AHT, OHT, and the BJC under both global warming and cooling conditions.

The CO_2_pulse and CDRMIP experiments could provide a clue on a climate reversibility, which is an ability for restoring toward its initial climate state^19–21^. The opposite case refers irreversibility. Irreversibility of a climate system could occur when a climate component passes a threshold such as ‘tipping point’; and/or when the response timescale of a climate component is relatively longer compared to a forcing timescale^22^. Such irreversible climate change usually accompanies a hysteresis behavior. For example, a strong hysteresis behavior of Atlantic Meridional Overturning Circulation (AMOC) could lead to an irreversible climate change^19,22,23^, and a strong oceanic thermal inertia over Southern Ocean could be a cause for irreversible climate change^21,24^. In this regard, the investigation of OHT gives a sense of where the irreversible climate change would occur by showing a place for the oceanic heat accumulation.

This study aims to investigate the following: (1) global climate response to abrupt increases and decreases in CO_2_ that would allow, to some extent, the reversibility of climate system to be addressed, as was the main purpose of the CDRMIP; (2) regional climate response, especially focusing converging and/or diverging of energies associated with general circulation and meridional heat transport; and (3) transient response of AHT and OHT. In “Model and experimental design”, the model and experimental design are introduced. The main results are presented in “Results”. Concluding remarks are given in “Discussion”.

## Model and experimental design

In this study, a 4 × CO_2_ pulse experiment (hereafter 4 × CO_2_PLS) was performed using the Community Earth System Model version 1.2^25^. This model is composed of atmosphere with an approximately 1° × 1° horizontal resolution and 30 vertical levels (The Community Atmospheric Community Atmospheric Model version 5)^26^; ocean with an approximately 1° × 0.3° resolution near the equator, with a gradual increase to 0.5° near the pole and 60 vertical levels (The Parallel Ocean Program version 2)^27^; sea ice (The Community Ice Code version 4); and land surface including the carbon–nitrogen cycle (Community Land Model version 4)^28^.

**Figure 1:**
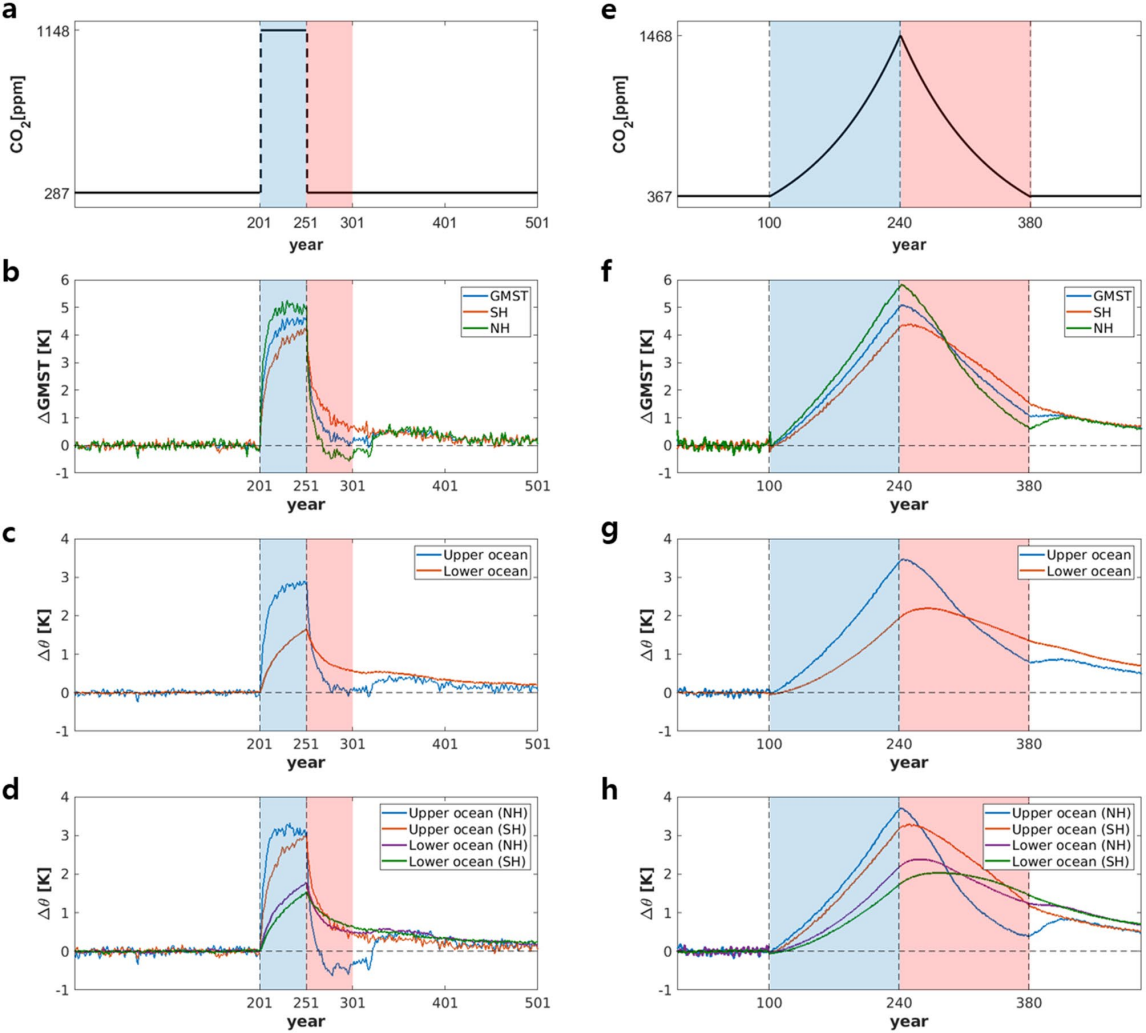
Time series obtained from 4 × CO_2_PLS experiment for (**a**) atmospheric CO_2_ concentrations, (**b**) deviations in the global mean surface air temperature (GMST) and hemisphere-averaged surface air temperature, (**c**) deviations in global mean potential temperature in the upper and lower oceans, (**d**) deviations in hemisphere-averaged potential temperature in the upper and lower oceans, from corresponding means of the pre-industrial runs. (**e**–**h**) As in (**a**–**d**), respectively, for 4 × CO_2_CDR experiment. Blue and red shaded portions indicate 50-year periods when forcing was switched on (ON50Y) and off (OFF50Y) for (**a**–**d**), respectively, and ramp-up and ramp-down periods for (**e**–**h**), respectively. Units are represented on the scales in each panel.

In the 4 × CO_2_PLS experiment, CO_2_ levels four times that of the PI level (1148 ppm) was switched on for 50 years and then immediately switched off to allow PI levels (287 ppm) to be reached again. Integral durations for the PI perpetual, 4 × CO_2_ forcing-on, and restoration periods were 201, 50, and 250 years, respectively (Fig. 1a). However, we focus mainly on the 50 years of the forcing-on period (years 202–251; hereafter, ON50Y) and the 50 years of the early restoration period (years 252–301; hereafter, OFF50Y). A period of 50 years is longer than a time scale for a fast response like the atmosphere yet sufficiently long enough to induce a slow response to global warming, such as that of deep ocean circulation^29^. Therefore, a 50-year mean can provide the spatial structure of the slowly evolving component.

A CDRMIP-type experiment (hereafter, 4 × CO_2_CDR) using the same model as 4 × CO_2_PLS was also performed. For 4 × CO_2_CDR, the atmospheric CO_2_ concentration was increased by 1% annually until it reached four times the present-day (PD) level (367 ppm). This was followed immediately by a symmetric decrease until the CO_2_ concentration reached the PD level (Fig. 1d). Subsequently, a constant CO_2_ level (367 ppm) was applied for 220 years. This experiment included a total of 28 ensemble members; therefore, the results of this study as given in this study all refer to 28-member ensemble means.

To understand the restoration processes, we analyzed atmospheric and oceanic meridional heat transport. To determine the flow of heat, the system was divided vertically into atmospheric and upper- and lower-oceanic layers in terms of global general circulation. The range of the ocean was set to be global and from the surface to a depth of 2000 m to reflect the influence changes in global overturning circulation and exclude the effects of topography. Since the oceanic response below 2000 m is very weak, the change in below 2000 m was excluded in our analysis. The upper and lower oceans in this study were separated by a fixed mixed-layer depth (MLD). Here, the MLD was defined as the shallowest depth where the local, interpolated buoyancy gradient matched the maximum buoyancy gradient between the surface and any discrete depth within the water column^30^. As the overall MLD changed with CO_2_ forcing, the MLDs at each grid for the entire experimental period were fixed as those obtained from the PI or PD data for the 4 × CO_2_PLS or 4 × CO_2_CDR experiments, respectively. All data used in this study are annual mean quantities.

## Results

### Global climate change

We first analyzed the global climate response in the 4 × CO_2_PLS and then compared it with the results from the 4 × CO_2_CDR. By switching on CO_2_ forcing in 4 × CO_2_PLS, the GMST increased abruptly, up to 4 °C, within 10 years and rather slowly, by 0.5 °C, for the remaining 40 years (Fig. 1b). After CO_2_ forcing was turned off, GMST decreased quickly, by 1 °C within 3–4 years–which corresponds to an initial fast exponential-type decay with an e-folding time less than 5 years^29^–before then decreasing at a slower pace. However, for 250 years, the GMST was slightly warmer compared to PI condition. The mean surface temperatures over the Northern Hemisphere (GMST only in the NH) and Southern Hemisphere (GMST only in the SH) show behavior similar to the GMST, except that the GMST in the NH was warmer than the GMST in the SH during the forcing-on period; the opposite was true during the early forcing-off period. In other words, there were abrupt asymmetric changes in hemispheric warming/cooling between the forcing-on and forcing-off periods. This hemispherically different response is primary caused by an hemispheric asymmetry in heat capacity associated with different land-mass distribution.

The change in the global-averaged upper ocean temperature (GUOT) was almost identical to that of the GMST, but the maximum temperature of the former was approximately 3 °C (Fig. 1c). The global-averaged lower ocean temperature (GLOT) increased almost linearly during the forcing-on period (Fig. 1c). The maximum increase in the GLOT was about 1.7 °C. After the forcing-off occurred, the GUOT decreased abruptly, similar to what happened with the GMST, while the GLOT decreased slowly. The GUOT reached the PI level rather quickly before increasing again toward a condition slightly warmer than the PI level. The GUOT was directly influenced by surface heat fluxes. However, because of its higher capacity to retain heat, its change in temperature was weaker than that of the land surface; therefore, the maximum GMST was larger than the maximum GUOT. However, GLOT was controlled by processes related to ocean dynamics, which are slow response components; therefore, changes in temperature show strong thermal inertia.

The ocean temperature response is different in the Northern Hemisphere (NH) and the Southern Hemisphere (SH) (Fig. 1d). During the forcing-on period, GUOT in the NH quickly increased to reach a peak within 10 years and maintained this level with only a slight change. GUOT in the SH, however, increased slowly but continuously and reached a maximum at the end of the forcing-on period. Such a hemispheric difference in GUOT change is likely due to both the reduction in oceanic heat transport by the AMOC and the smaller area of ocean and greater area of land in the NH. Thus, although last two factors may have been the cause of relatively faster warming in the NH, the warming trend was disrupted by the reduced thermal advection of the AMOC. However, in the SH, a greater area of ocean with a high capacity to retain heat may have caused slow but continuous warming.

After the forcing was turned off, the GUOT in the NH decreased to levels even lower than those in the PI (Fig. 1d). This “overshooting” in a cold period was maintained for more than 50 years before quickly increasing to GUOT above PI levels. The GUOT in the SH also showed an abrupt cooling, but its rate of decrease was much less than that in the NH, which did not exhibit overshooting. After cooling, the GUOT in the SH merged with the GLOT, implicating that the stored heating in GLOT during forcing-on period transported to GUOT. Changes in the GLOT of the NH and SH were similar to each other, except that the former was slightly warmer during ON50Y and had a faster cooling trend during OFF50Y compared to the GLOT in the SH. As Fig. 1c,d shows, oceanic stratification was enhanced during the forcing-on period, i.e., the GUOT increased more than GLOT. This likely suppressed vertical mixing and/or convection, which resulted in a weakening of MOC, especially NH AMOC^19^ (Fig. 1d), consequently causing a following strong cooling of NH GUOT due to a weakening of oceanic heat transport. On while the strong cooling of the GUOT in the NH during the early forcing-off period reduced oceanic stratification, which gives a favorable condition for quick recover of AMOC. This peculiar behavior of the GUOT in the NH during the forcing-off period might be related to the meridional overturning circulation (MOC) in the ocean, as documented in the previous study on 4 × CO_2_CDR^19^, in which the relaxed oceanic vertical stratification (i.e., Fig. 1d,h) and enhanced meridional salinity gradient during a ramp-up period were proposed as the reason for a quick recovery of AMOC.

The 4 × CO_2_CDR produced results very similar to those from 4 × CO_2_PLS (Fig. 1f–h) although the scenarios of CO_2_ change are quite different (Fig. 1a.e). The maximum increases in GMST, GUOT, and GLOT in 4 × CO_2_CDR were slightly higher than those in 4 × CO_2_PLS, possibly because of the longer time for which CO_2_ forcing was applied. The slow cooling of GLOT in 4 × CO_2_PLS was also visible in 4 × CO_2_CDR (Fig. 1g), and the strong cooling of the GLOT in the NH and other changes in oceanic temperature in 4 × CO_2_CDR showed features quantitatively like those of 4 × CO_2_PLS (Fig. 1h).

**Figure 2:**
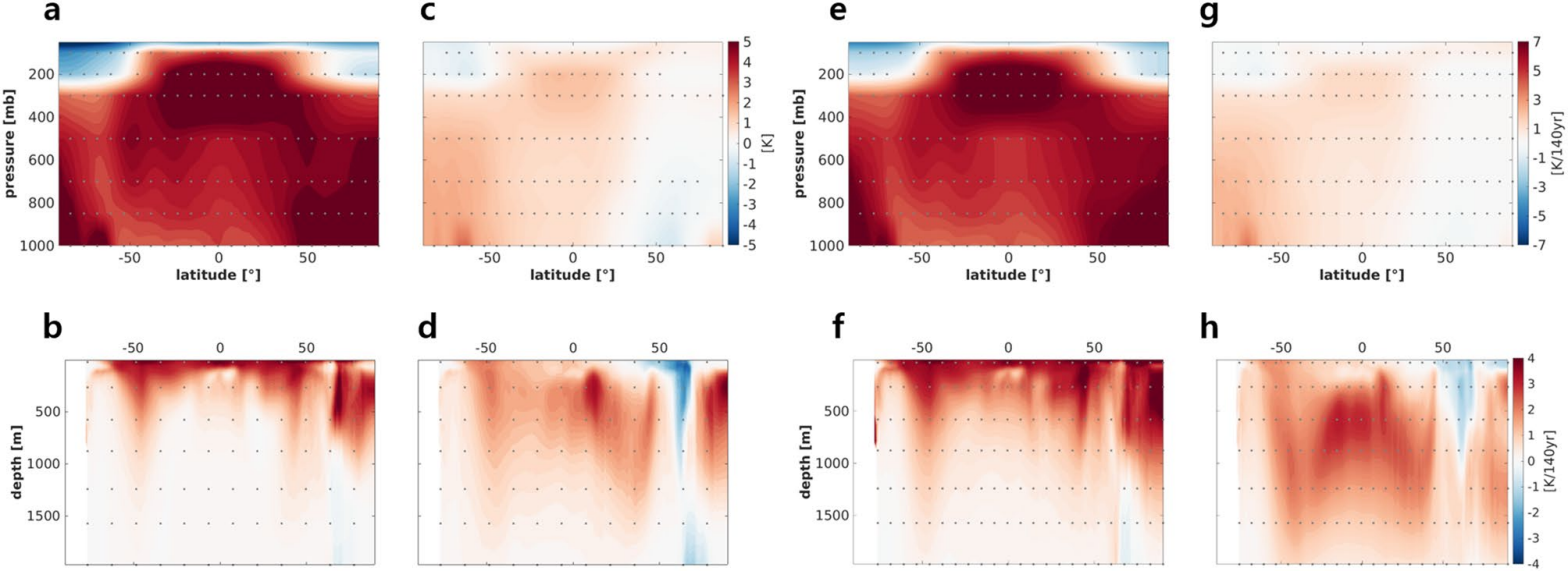
Time- and zonal-means of (**a**) atmospheric temperature and (**b**) oceanic potential temperature deviations from PI experiment in the ON50Y period. (**c**) and (**d**) As in (**a**) and (**b**), respectively, but for OFF50Y. Linear trends in (**e**) zonal-mean atmospheric temperature and (**f**) zonal-mean ocean potential temperature for the CO_2_ ramp-up period obtained from the 4 × CO_2_CDR experiment. (**g**) and (**h**) As in (**e**) and (**f**) but for sum of linear trends from the CO_2_ ramp- and -down periods, respectively. Units are given on the right-hand side of each panel. Dots in the figures indicate statistically significant points above 99% confidence tested by the bootstrap method.

To investigate local changes in temperature, we computed the deviations from zonal- and time-mean atmospheric and oceanic meridional temperatures during ON50Y and OFF50Y; the deviation indicated the difference from the mean of PI perpetual experiment. For ON50Y (Fig. 2a), tropospheric warming and stratospheric cooling were found; the strongest warming was observed in the upper troposphere over the tropics, as seen in observations of warming trends and in most coupled models^31–33^, and near the surface in polar regions. Strong surface warming in the polar region, the so-called ‘polar amplification’ was thought to be related to surface albedo feedback^34,35^, lapse rate feedback^36,37^, and enhanced poleward heat flux through the atmosphere^38^ and ocean^39^.

In the ocean, overall warming is dominant at the surface layer, which is directly affected by atmospheric warming, and its structure is a hemispherically symmetric (Fig. 2b). Surface warming signals penetrate to depths of approximately 1 km over the mid-latitudes (40°–60°) and approximately 300 m over subtropical regions in both hemispheres. Upper-to-lower ocean heat transport is related to the efficiency of vertical thermal diffusion, downwelling, and the change in meridional head advection. In the following sections we show a detail process.

Forcing-off led to a marked change in the patterns of warming and cooling. For OFF50Y (Fig. 2c), warming continued in the upper troposphere over the tropics and the troposphere over high latitudes in the SH, but its amplitude was reduced significantly. Weak cooling appeared in the near-surface temperature in the high latitudes in the NH. In the ocean, surface warming was strongly reduced worldwide except in the mid-ocean, at depths between 200 and 600 m (Fig. 2d). Relatively strong warming occurred at depths of 100–500 m in the tropical region of the NH, in particular, and there was strong cooling over the deep Arctic Ocean. The deviation for OFF50Y (i.e., [$\widetilde{OFF50Y}-\widetilde{PI}$], where the tilde indicates the time mean) in this study may be interpreted as a non-symmetric climate response in 4 × CO_2_PLS such that $\left[\widetilde{ON50Y}-\widetilde{PI}\right]+\left[\widetilde{OFF50Y}-\widetilde{ON50Y}\right]=[\widetilde{OFF50Y}-\widetilde{PI]}$, where the first and second terms of the left-hand side indicate the responses to increasing CO_2_ and decreasing CO_2_, respectively; thus, the residual from their sum can be assumed as to indicate an asymmetric climate response to CO_2_ forcing.

The linear trend during the ramp-up of CO_2_ forcing from the 4 × CO_2_CDR experiment (Fig. 2e,f) was quite similar to the difference between ON50Y and PI experiment (Fig. 2a,b). The sum of the linear trend for ramp-up and ramp-down periods (Fig. 2g,h) also resembled the difference between OFF50Y and PI experiment (Fig. 2c,d), except that the warming in the mid-ocean appeared much deeper in the ocean with a stronger signal. A deeper intrusion of a warming signals may also be related to the longer forcing period applied. As mentioned previously, these results represent an asymmetric climate response to symmetric CO_2_ forcing. This asymmetric climate response can be considered as a hardly recoverable or irreversible climate signal, which can presumably be attributed to the delayed response of slow-adjusting climate components or tipped climate elements. However, a clear dynamical interpretation of this process is beyond the scope of this study.

Although the comparison between the 4 × CO_2_PLS experiment and 4 × CO_2_CDR experiment was performed qualitatively, the resemblance between these two experiments during the phases when CO_2_ both increased and decreased is remarkable. Therefore, in the following sections, we will focus mainly on the 4 × CO_2_PLS experiment as it had some advantage in presenting an understanding of the fundamental behavior of climate systems under scenarios less complicated than those used in the 4 × CO_2_CDR experiment. Furthermore, to understand the structure of temperature change during the two periods, the associated changes in global atmospheric and oceanic general circulation are explored below.

### Atmospheric and oceanic general circulation

**Figure 3:**
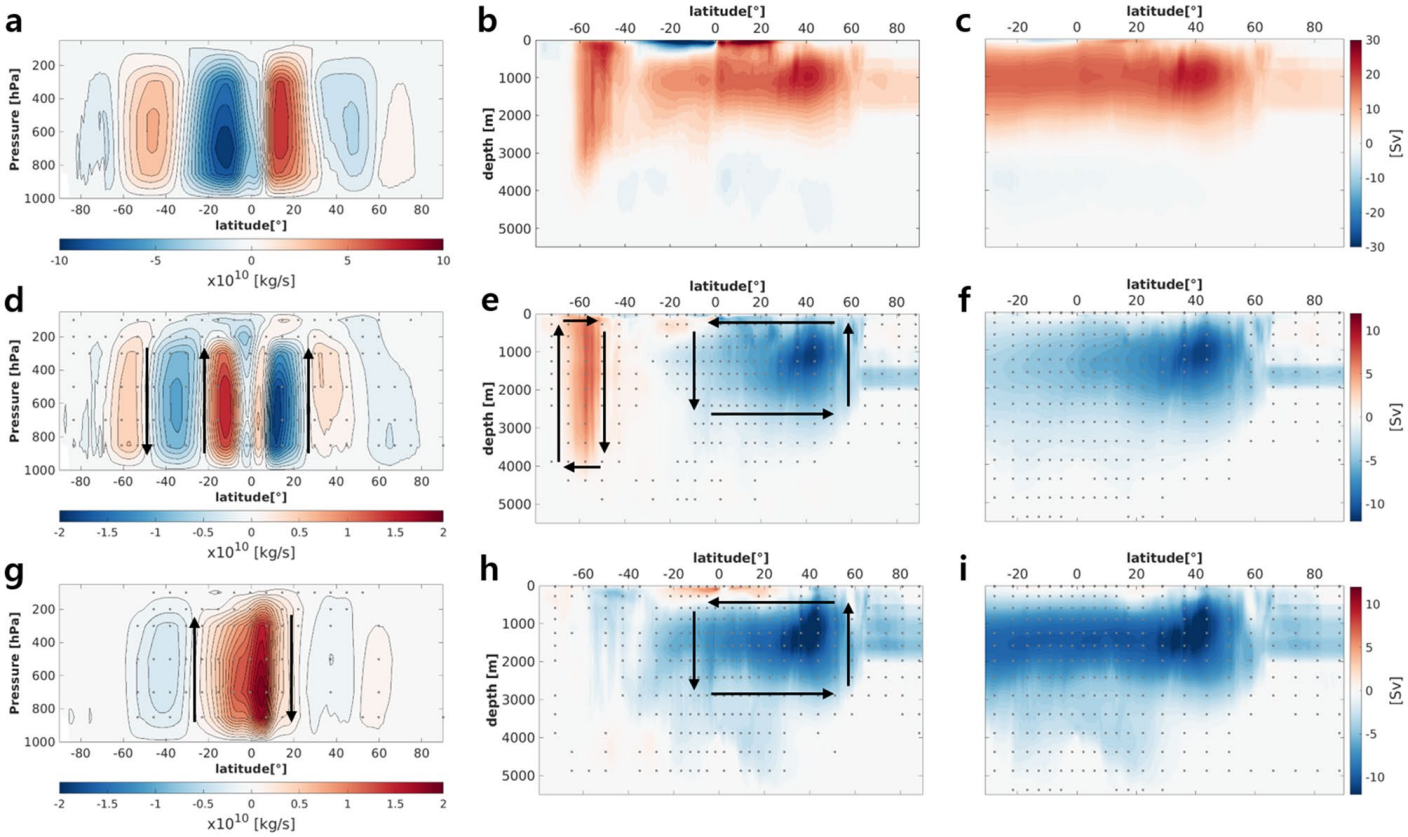
Vertical cross-section of annual-mean values for (**a**) atmospheric mean meridional circulation (MMC), (**b**) global meridional overturning circulation (MOC), and (**c**) Atlantic MOC obtained from PI experiment. (**d**) MMC, (**e**) global MOC and (**f**) AMOC for the ON50Y period as in (**a**–**c**), respectively, but for deviations from means of PI experiment. (**g**–**i**) as in (**d**–**f**), but for the OFF50Y. Units for the atmospheric MMC and oceanic MOC are 10^10^ kg^−1^ and are Sv, respectively. Arrows indicate the direction of circulation. Dots indicate grids where the deviations are statistically significant with 99% confidence, as tested using the bootstrap method.

As shown in Fig. 3a, the annual mean meridional circulation (MMC) of the atmosphere during the PI period (Eq. (1) in the “Method” section) is well simulated and shows that the Hadley circulation has a rising branch slightly north of the equator and sinking branches at the subtropics in both hemispheres, and that the Ferrel and polar cells in both hemispheres are well positioned as observed. Their oceanic counterparts (Fig. 3b; Eq. (2) in the “Method” section) features shallow tropical overturning circulations in both hemispheres, a deep MOC at depths of 500–2500 m over an area from 40° S to 60° N, and a much deeper mid-latitude MOC in an area from 40° S to 60° S (the “Deacon cell”)^40^. Focusing only on the Atlantic Ocean allows clear identification of the AMOC (Fig. 3c); the changes in the global MOC (Fig. 3b) are largely due to those in AMOC.

The MMC deviation in the ON50Y compared to PI experiment shows a weakening of the Hadley circulation^41^ and its widening in both hemispheres due to increased CO_2_ forcing^42–45^, as indicated by negative and positive streamfunctions around 35° S and 35° N, respectively (Fig. 3d). This weakening of the Hadley circulation might be related to an increase in the static stability (${S}_{p}$) of the atmosphere over the tropics^41^ inferred from vertical gradient of potential temperature (not shown here but very similar to Fig. 2a). In the tropics, diabatic heating (${Q}_{d}$) is primarily balanced with adiabatic cooling (i.e., adiabatic expansion of rising air; ${S}_{p}\omega$ where $\omega$ is the pressure velocity) over a long-term time scale^46^; therefore, a more stratified atmosphere suppresses upward motion (i.e., $-{S}_{p}\omega \approx \frac{{Q}_{d}}{{C}_{p}}; {C}_{p}$ is the specific heat of air). Similarly, an increase in the static stability of the subtropics pushes the baroclinic instability zone to poleward, likely leading to the poleward migration of the jet and the consequent setting of the poleward extent of the Hadley circulation^42,45,47^.

The deviation of the oceanic MOC in the ON50Y strongly weakened over the whole NH and slightly weakened over low latitude of SH (Fig. 3e). The weakening of the AMOC was particularly prominent over NH (Fig. 3f) and was a common feature in climate model simulations under global warming scenarios^48,49^. The Deacon cell was enhanced slightly, and its center moved slightly southward; this was associated with the southward shift of the surface westerly that drove it. The southward shift of the surface westerly was also related to the southward migration of the Ferrel cell.

During the OFF50Y period, the change in the Hadley cell with respect to the PI era was identified as an enhancement in the northern cell and a reduction in the southern cell; this also indicated a southward shift in ITCZ (Fig. 3g). Such changes in the Hadley cell and ITCZ were likely associated with a hemispheric contrast in changes in atmospheric temperature (Figs. 1b and 2c). To compensate for the heat imbalance between the NH and SH in an anomalous thermal distribution, cross-equatorial heat transport toward the NH was induced and led to a southward shift in the ITCZ^50^. In the ocean, the shallow overturning cell in the tropics changed asymmetrically in both hemispheres–becoming enhanced in the NH and reduced in the SH (Fig. 3h)–in association with the enhanced NH and suppressed SH Hadley cell. However, the deep ocean circulation in the OFF50Y period was suppressed even further and expanded to SH compared to its state in the ON50Y period, mostly due to the suppression of the AMOC (Fig. 3i). This excessive suppression of the AMOC (i.e., the “overshoot” of the AMOC) has been seen in similar experiments that included scenarios in which CO_2_ levels were changed^23,51,52^ and in the 4 × CO_2_CDR experiment^19^. Such studies argued that this overshooting of the AMOC (Fig. 3i) was related to the salinity that accumulated in the subtropical Atlantic Ocean during the period when CO_2_ concentrations were high, as this enhanced a salt advection feedback and led to further reductions in the AMOC, and a relaxed vertical stratification that made oceanic convection easier during the period when the CO_2_ levels changed. A slowdown in the AMOC led to cooling at the surface of the Atlantic ocean in subarctic to Arctic regions and warming in the deep ocean around 50° N because of less cold surface water was intruding into that area (Fig. 2d). A slight reduction in the Deacon cell might possibly be related to the weakened surface winds associated with a reduction in the meridional surface temperature gradient (Fig. 2c,d) because the Deacon cell is driven mainly by wind-driven upwelling.

So far, we have described the changes in the meridional atmosphere and ocean thermal structures in the ON50Y and OFF50Y periods and their dynamic links with meridional atmospheric and oceanic circulations. Initially, the thermal structure and its response were determined by the local energy balance. However, the long-term balance in a local climate can be determined through energy transport. In the following section, we discuss meridional heat transport and its role on local energy balances.

### Meridional heat transport

**Figure 4:**
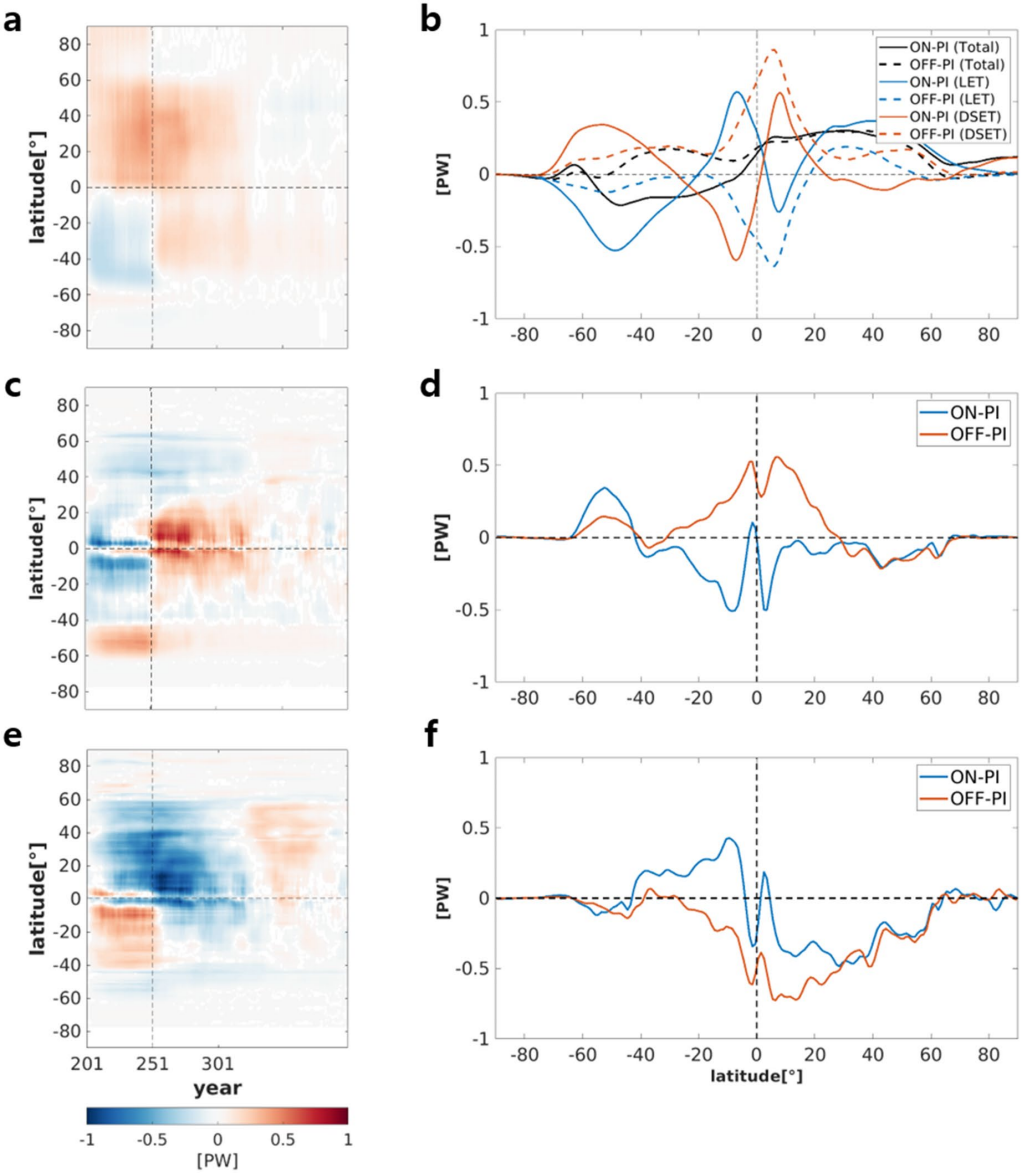
Hovmöller diagram of zonally-averaged (**a**) atmospheric heat transport (AHT), (**c**) upper oceanic heat transport (OHT) and (**e**) lower OHT deviations from the mean of PI experiment. Differences in time-mean for (**b**) AHT, dry static energy (DSET), and latent energy transport (LET), (**d**) upper OHT and (**f**) lower OHT between the ON50Y (OFF50Y) and mean of PI experiment. Units are Petawatts. ON and OFF indicates ON50Y and OFF50Y, respectively.

As forcing was turned on in 4 × CO_2_PLS, the poleward heat transport from the equator to high latitudes via AHT (Eq. (3) in the “Method” section) increased in both hemispheres (Fig. 4a). The poleward heat transport from the subtropics to mid-latitudes in both hemispheres (between 20° and 60°) was due to the increase in latent heat transport, in which the poleward transport of latent heat ware larger than the equatorward transport of dry static energy (Fig. 4b). This was a result of the increasing specific humidity associated with increasing air temperature and the poleward expansion of the Hadley circulation. Here, the moist static energy was defined as $m={C}_{p}T+gz+Lq$, where *T* is the temperature, ${C}_{p}$ is the specific heat at a constant pressure, *z* is the height, *g* is the gravitational acceleration, *L* is the latent heat of vaporization at 0 °C, and *q* is the specific humidity. There was an enhancement in convergence of the latent heat ($Lq$; LET) in the region of the tropics to the equator; however, the divergence of dry static energy (${C}_{p}T+gz$; DSET) associated with the weakened Hadley circulation overcompensated for the convergence of the latent heat thus resulting in poleward AHT in the tropics as well (Fig. 4b).

Immediately after the CO_2_ forcing was turned off in 4 × CO_2_PLS, the AHT became positive for the region from 50° S to 60° N, indicating an enhancement in heat transport from the SH to the NH. The northward AHT over the tropics was mainly driven by the dry static energy, while the latent heat transported to the south. This northward AHT in the tropics was related to a clockwise overturning circulation response in the region (Fig. 3g). The counterclockwise circulation responses over the mid-latitude regions in both hemispheres (Fig. 3g) also contributed to the positive AHT. Such hemispheric energy transport reduced the hemispheric thermal contrast between the relatively warmer SH and colder NH (Fig. 2c). The hemispheric energy transport in the atmosphere decayed slowly as the thermal contrast was reduced (Fig. 4a).

During the forcing-on period, an overall southward transport was observed in OHT in the upper ocean in the tropics to mid-latitudes in both hemispheres, although the transport in NH was weaker than that in SH (Fig. 4c,d); this was related somewhat to the slowdown in the AMOC and Hadley circulation. The northward OHT appeared between 40° S and 60° S, thus showing that there was a convergence of heat at approximately 40° S (Fig. 4d). During the forcing-off period, northward upper OHT was dominant in the tropics, presumably associated with the strong clockwise atmospheric overturning circulation response (Fig. 3g). In the mid-latitudes, southward upper OHT and northward upper OHT were observed in the NH and SH, respectively. These may have been related to the counterclockwise atmospheric overturning circulation responses in the mid-latitude regions of both hemispheres.

As shown in Fig. 4e,f, there was an equatorward convergence of the lower OHT during the forcing-on period that corresponded to a weakening of the AMOC, particularly in the NH, and a weakening of the MOC in the SH. Southward lower OHT was dominant over tropical area in both hemisphere and midaltitude in the NH during the forcing-off period, which is opposite to the trend in the AHT and upper OHT; this was likely associated with the global-scale weakening of the AMOC. Such an inverse relationship between AHT (and upper OHT) and lower OHT may be interpreted as a total heat transport constraint (i.e., the BJC); OHT induced by the AMOC tended to be opposite to the AHT in a low-frequency band while wind-driven circulation such as the oceanic gyre-induced upper OHT, tended to be in-phase with the AHT^18^.

## Discussion

In this study, we investigated global-scale climate change, including thermal structures and atmospheric and oceanic meridional overturning circulations/thermal energy transport obtained from 4 × CO_2_PLS and 4 × CO_2_CDR experiments. First, we found that 4 × CO_2_PLS and 4 × CO_2_CDR produced qualitatively similar results although they used different scenarios related to changes in CO_2_ forcing. This suggested that the climate response in 4 × CO_2_PLS could be analogous to the transient climate response in 4 × CO_2_CDR. In 4 × CO_2_PLS, the abrupt return to 1 × CO_2_ led to hemispherically asymmetric cooling, i.e., more cooling over the NH, which enhanced the Hadley cell in the NH and suppressed it in the SH, thereby enhancing northward AHT. Furthermore, the slow and delayed response of the AMOC to CO_2_ forcing led to a further reduced deep ocean meridional circulation during the period of the abrupt return to 1 × CO_2_ as compared to the abrupt 4 × CO_2_ forcing period. Therefore, southward OHT is enhanced in the lower ocean, may have enhanced the oceanic thermal memory effect in the SH, and might be related to the relatively slow return to PI condition of SH lower ocean temperature.

In recent decades, north Atlantic warm holes that have been suggested as being related to reductions in OHT associated with the slowdown of the AMOC have been observed in the north Atlantic^48,53^. Furthermore, a recent study^54^ also demonstrated the important role of AMOC slowdown in the formation of North Atlantic warm hole under the higher concentration CO_2_ scenario (i.e., Representative Concentration Pathways 8.5). The southward OHT in NH was also observed during the forcing-on period in the 4 × CO_2_PLS experiment (Fig. 4f). This southward OHT was even stronger during the early forcing-off periods and was associated with a further reduction in the AMOC. In the tropics, southward OHT in the lower ocean was largely compensated by northward OHT in the upper ocean (Fig. 4d), while in mid-latitudes the northward AHT was dominant (Fig. 4b). The cause of the northward AHT is unclear; however, the northward AHT in mid-latitudes NH may lead to warming over land.

## Method

### Computation of meridional circulation

In the atmosphere, the MMC on the pressure coordinate is expressed as a mass stream function, that is:

1 $${\Psi }_{a}\left(z,\phi \right)=\frac{2\pi {a}_{e}}{g}{\int }_{p}^{{p}_{s}}{v}_{a} cos\phi dp,$$

where p, ${\mathrm{p}}_{\mathrm{s}}$, ${a}_{e}$, $\phi$, g, and ${v}_{a}$ are the pressure, surface pressure, Earth’s radius, latitude, gravitational acceleration, and atmospheric zonal-mean meridional wind, respectively. Similarly, the MOCs of the ocean, including the Eulerian and eddy-induced ones, are obtained by the following equation:

2 $${\Psi }_{o}\left(z,\phi \right)={\int }_{{\lambda }_{E}}^{{\lambda }_{W}}{\int }_{z}^{0}{v}_{o}cos\phi dzdx,$$

where z, ${\lambda }_{E}, { \lambda }_{W}$ and ${v}_{o}$ ($={v}_{Euler}+{v}_{Eddy}$) indicate the vertical depth of the ocean, longitudinal boundaries, and total oceanic meridional velocity, respectively.

### Computation of meridional heat transport

Meridional AHT is computed using an energetic constraint^55^. The energetic constraint indicates that the total energy transport must act to maintain a local energy balance^56^; therefore, the zonal-mean net heating of the atmosphere, ${Q}_{net}$, must be balanced via the divergence of northward AHT on longer time scales^54^:

3 $${Q}_{net}\left(x\right)=\frac{1}{2\pi {{a}_{e}}^{2}}\frac{dF}{dx}$$

where $x=\mathrm{sin}(\phi )$ and $\phi$ is the latitude. ${Q}_{net}$(= ${Q}_{TOA}-{ Q}_{sfc}$) denotes the difference between the net heat flux at the top of the atmosphere and that on the surface of the land. Taking the meridional integral of ${Q}_{net}$ yields the northward AHT:

4 $$F\left(x\right)=2\pi {a}^{2}{\int }_{-1}^{x}{Q}_{net}\left({x}^{*}\right)d{x}^{*}$$

The AHT computed using a traditional method such as dynamic process produces results very similar to those computed by energetic constraints^55^. The meridional OHT was computed using dynamical processes as follows:

5 $$F\left(x\right)=2\pi a{(1-{x}^{2})}^{1/2}{c}_{p}\rho {\int }_{{H}_{1}}^{{H}_{2}}[\theta {v}_{o}]dh$$

where ${c}_{p}$ is the specific heat, $\rho$ is the density of the sea water, $\theta$ is the ocean potential temperature, and the integral over depth *h* is from H_1_ to H_2_. For the upper ocean, H_1_ and H_2_ are the MLD and surface, respectively; for the lower ocean, H_1_ and H_2_ are 2000 m and MLD, respectively. It should be noted that OHT computed by using Eq. (5) is sensitive to the choice of temperature scale. Here, Celsius unit was used. The amplitude of OHT with Kelvin unit was larger than the current result, yet two results were qualitatively very similar (not shown here). Thus, the qualitative based interpretation is acceptable.

## Data Availability

The data used in this study are available at https://data.mendeley.com/datasets/pngpktkvm6/3.
